# Addictive Behavior and Evolutionary Adaptation: Mitigated through Genetic Addiction Risk Severity Early Identification and Awareness Integration Theory

**DOI:** 10.18103/mra.v12i8.5702

**Published:** 2024-08-30

**Authors:** Foojan Zeine, Nicole Jafari, Eileen Manoukian, Kenneth Blum

**Affiliations:** 1 Awareness Integration Institute, San Clemente, CA. 92672, USA.; 2 Department of Health Science, California State University at Long Beach, Long Beach, CA., 90804, USA; 3 Department of Applied Clinical Psychology, The Chicago School of Professional Psychology, Los Angeles, CA., 90017, USA; 4 Global Growth Institute, San Clemente, CA. 92672, USA; 5 GemEducare, Tarzana, CA. 91356, USA; 6 Department of Psychiatry, University of Vermont, Burlington, VT., 05405, USA; 7 Center for Sports, Exercise, Mental Health Western University Health Sciences, Lebanon, OR., USA; 8 Department of Molecular Biology and Adelson School of Medicine, Ariel University, Ariel, Israel; 9 Institute of Psychology, ELTE Eötvös Loránd University, Budapest, Hungary

## Abstract

**Objectives:**

Humans, with their unique genetic profile, exhibit a greater propensity to develop and maintain addiction compared to other animals. This paper offers a detailed examination of addiction, co-occurring traits, and psychologic disorders, focusing on neurobiological and molecular aspects. Furthermore, the authors investigate the potential of the Awareness Integration Theoretical model as an effective therapeutic addiction treatment.

**Methods:**

Using PsychINFO, PubMed, and Google Scholar, a comprehensive literature review was conducted on the evolutionary and adaptation pathways to addiction, epigenetic factors, and the potentiality of Awareness Integration Theory in treating addiction.

**Results:**

Epigenetics allows environmental factors to create lasting and heritable phenotypic changes, enabling rapid adaptation to these stimuli. Addiction “high-jacks” this system and the neurochemical mechanisms that control flexibility and innovation and is, thus, the price we pay for adaptability. Drug addiction is thought of as an adjunctive behavior or a subordinate behavior catalyzed by more profound, more significant psychological and biological stimuli.

**Conclusions:**

The neurochemical mechanisms underlying addiction, a complex interplay of genetic and environmental factors, are intertwined with the hallmark features of the human species, such as behavioral flexibility and pre-addictive propensity. The dopaminergic system, a key player in addiction, serves as a crucial link between addiction and the shared genetic profile evident in co-occurring traits and psychiatric and psychological disorders. Furthermore, a hypofunctioning dopaminergic system is a common characteristic of addiction and co-occurring psychiatric and psychological disorders. Early childhood preventative measures are vital in re-directing the existing predictive and poor adaptability functioning, which refers to the individual’s inability to adapt to changing circumstances and reliance on maladaptive coping strategies. Awareness Integration Theory’s approach encompasses a therapeutical model addressing individuals’ physical, cognitive, and psychosocial domains, allowing the individual to address intergenerational and ancestral ineffective and harmful adaptability. This, in turn, AIT will allow the human genome to be on a healthier path to recovery from obstacles such as addiction. When a tendency or a characteristic improves your ability to function and survive, and especially your ability to produce and raise children, that will most likely break the cycle of addiction and addictive behavior.

## Introduction

The evolutionary perspective on addiction suggests that addictive behaviors may have offered advantages to our ancestors, which contradicts the contemporary view that addiction is purely maladaptive. For example, substances like alcohol may have provided survival benefits such as fueling the body with calories or promoting the healing process through its medicinal properties. Regardless, addiction is a complex phenomenon influenced by genetic, psychological, social, and environmental factors. It is well-known that a gene variant in the brain reward circuit, the dopamine D2 receptor A1 gene, caused significantly reduced dopamine D2 receptors, possibly 47 million years ago, provided risk-taking behavior and hunter-gatherer benefits^1^. While some addictions may have been advantageous in ancestral contexts, in modern times, the ease of access to highly addictive substances or engagement in addictive activities can pose significant risks that could lead to harmful outcomes.

The recognition that addiction is deeply rooted in human evolutionary history offers a growing paradigm of thought and study. The scientific community has acknowledged that addictive plant alkaloids have evolved to defend against insect herbivory. This suggests that addiction’s origins in fundamental biological mechanisms have been shared across taxa since early evolution. Based on this perspective, addiction is an invertebrate phenomenon that has humans caught in a coevolutionary struggle^2^.

Traditionally, drug addiction has been seen as a response to deeper psychological needs and biological stimuli rather than just a reaction to chemicals. Addiction to drugs serves as a compensatory response to a decrease in Darwinian fitness. It has been found to involve various issues in developmental attachment, pharmacological mechanisms, and social factors like inequality and dependence^3^.

In this paper, we propose repetitive behaviors triggering dopamine release, which initially reinforced motivation and actions vital for survival and reproduction in past resource-limited environments, may have evolved as adaptive mechanisms. However, in today’s world of abundantly rewarding stimuli, these exact mechanisms can lead to addictive behaviors and reward deficiency syndrome (RDS). Thus, in this context, addiction is viewed as an evolutionary byproduct stemming from the mismatch between ancestral and modern environments. It is important to understand that our behaviors are tied to both DNA antecedents or polymorphisms that set an individual up for survival (ancestral trait) and Chromosomal/histone as a function of the modern environment. In this scenario, a negative environment induces methylation onto histone for any particular gene with concomitant attenuation of mRNA transcription (expression).

In contrast, positive or reinforcing environmental stimuli (environmental state) augment mRNA transcription (expression) via acetylation onto histones for any particular gene. So, this evolutionary adaptive by-product related to addictive behaviors in the modern world is regulated by these known epigenetic chemical outcomes. Finally, the coupling of genes (trait) and environment (state) equally contributes to any adaptive behavior, including addiction. This points to the idea that any treatment plan must work on both sides of the coin^4^.

## Addiction

The American Psychological Association (APA) defines addiction as a state of psychological and/or physical dependence on substances like alcohol, as well as on specific behaviors such as sex, exercise, and gambling. Substance use disorder (SUD) encompasses physiological, behavioral, and cognitive symptoms linked to persistent substance use despite resulting problems, including impaired control and risky behaviors^5^. It is crucial to distinguish between different levels of involvement with substances or behaviors ranging from basic use, misuse, and SUD to full-blown addiction. While the initial engagement with psychoactive substances or behaviors that triggered dopamine release might have conferred an evolutionary advantage, the development of psychological attachment and physical dependence on these substances or behaviors in our current lives represents a deviation from successful adaptation that potentially leads to harmful outcomes. We are compelled to argue that while our ancestors and early *homo sapiens* evolutionarily relied on strong dopamine release to assist in defense, hunter-gathering, selfishness, etc., in modern times, high dopamine (hyperdopaminergia) release seen in adolescence developmentally augments the euphoric response of powerful psychoactive drugs like opioids, cocaine, cannabis, and alcohol as well as addictiveness to palliative foods^6^.

Moreover, in today’s times, it is now known and accepted worldwide that dopamine dysregulation is indeed a key to all mental diseases. This latter statement is supported by the overwhelming evidence in the literature showing explicit common dopaminergic mechanisms shared by most psychiatric disorders^7^. In 1995, one of us coined “Reward Deficiency Syndrome (RDS). “Reward Deficiency Syndrome,” a term first coined by Kenneth Blum in 1995, can be defined as: “A brain reward genetic dissatisfaction or impairment that results in aberrant pleasure-seeking behavior that includes drugs, excessive food, sex, gaming/gambling and other behaviors.” Based on overwhelming literature coupled with our recent GWAS and PGX work, we suggest “Reward Deficiency” as a more general term encompassing the nosology of “preaddiction”^8^. Such conceptualization offers immediate benefits in the form of early screening to detect high-risk individuals through the Genetic Addiction Risk Severity (GARS)^9^ test and the Reward Deficiency Syndrome Questionnaire.

The following section will present evolutionary, genetic, physiological, psychological, and societal factors suggesting that addiction is a byproduct of evolutionary adaptive behaviors.

## Evolutionary Factors

Addiction is a subordinate behavior that is not isolated to a pharmacologically and chemically induced state, but it is also aroused from more profound psychological and biological stimuli^10,11^. Several components can contribute to this arousal, such as genetic tendencies, early childhood adverse experiences, developmental attachment, parental care, or lack thereof, which may all be determinant factors leading to vulnerability to drug addiction. From an evolutionary perspective, children who receive more erratic care may focus more on short-term risks, a quality that could have been adaptive for survival in ancient environments. The use of addictive substances has falsely been attributed to the common belief that many substances of abuse have the superpower to heal and eliminate emotional pain that otherwise the person has to process and make sense of, resulting in a falsified sense of well-being and contentment, even if temporary^12^.

An evolutionary lens on drug use and addiction prompted two key inquiries that complement conventional medical viewpoints. First, what motivates humans to seek and consume non-nutritional substances repetitively? Secondly, why do the plants that are the primary sources of these substances produce chemicals capable of altering how the human nervous system functions? The following paragraphs related to evolutionary factors address these questions.

Addiction emerges from substances interacting with ancient reward systems designed to enhance behaviors that promote fitness in ancestral environments. This perspective also illuminates human distinctiveness, including the influence of cumulative culture and gene-culture co-evolution on behavior^13^. Rooted in survival instincts, these primary habitual behaviors involve pleasurable engagement with biological needs of food and sex and psychosocial fulfillment found in attachment. Cravings for these substances and activities drive these engagements, which are vital for the species’ survival. Secondary addictive behaviors arise when other pleasurable substances or activities become reliably available, often engaging innate reward systems more intensely than primary behaviors.

Viewed as supplementary or secondary behaviors triggered by more profound psychological and biological stimuli, drug addiction compensates for a decline in Darwinian fitness. It encompasses developmental attachment, pharmacological processes, and social factors like inequality, reliance, and dependence on others. As an example, evolutionarily speaking, children experiencing inconsistent care may prioritize short-term risks, which were advantageous in ancient environments. Recent work from Blum’s group found that while the x chromosome moderates nurture, they chromosome medicates risk-taking. The brain’s dopamine system biologically adapts to substance intake, predisposing individuals to addiction fueled by beliefs in substances’ healing properties. From an evolutionary perspective, drug use provides temporary benefits tied to ancient neural pathways shared by mammals. This often distorts perception, creating a sense of increased fitness and vitality driven by chemical changes interpreted as emotions and shaping human behavior^14^.

Ancient cultures didn’t perceive psychoactive plants as substances altering internal balance but as vital food sources with various benefits. Humans observed effects like tolerance to temperature changes, increased energy, and reduced fatigue, enabling longer foraging sessions and better survival practices during resource scarcity. Instead of being used for recreational purposes, these plants were valued for their nutritional content because they were rich in vitamins, minerals, and proteins. In environments with limited resources, mammals likely sought psychotropic chemicals as substitutes for scarce precursor nutrients in food sources, making drugs essential for preventing starvation and maintaining fitness. Early hominid species likely co-evolved alongside psychoactive plants, leading to civilizations eventually utilizing them for nutrition, enhancing their fitness and viability.

Psychoactive plants, over time, evolved to produce chemicals that deter threats from herbivores and pathogens. While not as potent as modern abused substances, these chemicals influenced the development of the mammalian central nervous system (CNS). They did so by mimicking mammalian neurotransmitters to disrupt normal CNS functioning, which indicated a co-evolutionary relationship between mammalian brains and psychoactive plants. This theory suggests an ecological interaction and mutual evolutionary response, wherein changes occur between mammalian brains and psychoactive plants, allowing them to influence each other’s evolutionary processes. The mammalian body, in response, has evolved defenses against excessive toxicity, such as metabolizing external substances and triggering vomiting reflexes^3^.

## Genetic Factors

In 1990, Blum, Noble, and their colleagues made a groundbreaking discovery by identifying the first gene polymorphism associated with severe alcoholism. Their blind experiment revealed that the presence of the A1 allele of the dopamine D2 receptor gene accurately classified 77% of subjects with alcohol use disorder (AUD), while its absence classified 72% of subjects without AUD. This polymorphic pattern, located on chromosome 11’s q22-q23 region, suggests a genetic influence on alcoholism susceptibility^15^.

Blum and Noble highlighted that the D RD2 A1 allele is linked not only to alcoholism but also to reward mechanisms. In 1995, they introduced reward deficiency syndrome (RDS) to describe behaviors associated with dopaminergic system dysfunction, which affects brain reward mechanisms. RDS behaviors include both substance (like cocaine) and nonsubstance addictive behaviors (such as gaming and pathological gambling), and they are characterized by dopamine-releasing dysregulation in the brain’s reward circuitry. In 1995, Blum et al. reported a predictive value of 74.4% using Bayes’ theorem, indicating that carriers of the DRD2 A1 allele were highly likely to exhibit various RDS behaviors, whether substance-related or not^15^.

## Dopaminergic Genes from an Evolutionary Perspective

The importance of dopaminergic signaling in evolution is reflected by the fact that dopamine modulates movement and behavior in species as diverse as worms and man, and dopamine receptors have been identified in various invertebrate and vertebrate species. In *Drosophila*, cDNAs encoding D1-like and D2-like dopamine receptors have been characterized^16,17^. Although the *Drosophila* polypeptides are considerably divergent in sequence from their mammalian counterparts, they exhibit the signaling and pharmacologic properties characteristic of mammalian D1 - and D2-like dopamine receptors^18,19^. Several D2-like dopamine receptors have been cloned and characterized by teleosts. For example, in the pufferfish *Fugu rubripes*, genes encoding the D2 and D3 receptor subtypes have been identified^20^.

Dopamine receptors are integral membrane proteins whose endogenous ligand is dopamine. They play a fundamental role in the central nervous system, and dysfunction of dopaminergic neurotransmission is responsible for the generation of a variety of neuropsychiatric disorders. From an evolutionary standpoint, phylogenetic relationships among the DRD_1_ class of dopamine receptors are still a matter of debate as, in the literature, different topologies have been proposed.

In contrast, phylogenetic relationships among the DRD_2_ group of receptors are well understood. Understanding the time of origin of the different dopamine receptors is also an issue that needs further study, especially for the genes with restricted phyletic distributions (e.g., DRD_2l_ and DRD_4rs_). Opazo et al. recovered the monophyly of the two groups of dopamine receptors^21^. The DRD_1_ clade was recovered sister to the aforementioned clade, and the group containing DRD_5_ receptors was sister to all other DRD_1_ paralogs. In agreement with the literature, among the DRD_2_ class of receptors, DRD_2_ was recovered sister to DRD_3_, whereas DRD_4_ was sister to the DRD_2_/DRD_3_ clade. According to our phylogenetic tree, the DRD_2l_ and DRD_4rs_ gene lineages would have originated in the ancestor of gnathostomes between 615 and 473 mya. Conservation of sequences required for dopaminergic neurotransmission and small changes in regulatory regions suggest a functional refinement of the dopaminergic pathways along evolution and subsequent adaptation.

## Reward Gene Variations in Human Social Networks

Evolutionary in humans, dominance has been linked to heritable personality traits, and superior status interacts with multiple neurotransmitter systems, such as DA D2/D3 receptor binding^22^. High binding is associated with higher social status, indicating the existence of biological systems. It can be automatically and efficiently inferred, indicating the existence of biological systems that process social rank or social hierarchy’s information^23,24,25^. A study by Zink et al. provides a characterization of the neural correlates associated with processing social hierarchies in humans Using fMRI, even in the absence of explicit competition, Zink et al. demonstrated that brain responses to superiority and inferiority are dissociable, both when encountering an individual of a particular status and when faced with an outcome that can affect one’s current position in the hierarchy^26^. They found that viewing a superior individual differentially engaged perceptual-attentional, saliency, and cognitive systems, notably the dorsolateral prefrontal cortex. Furthermore, social hierarchical consequences of performance were neurally dissociable and of comparable salience to monetary reward, providing a neural basis for the high motivational value of status. This work underscores the importance of hierarchy status in social networks, linking status to reward circuitry, a site of emotion and well-being.

In fact, according to Christakis and Fowler, people’s happiness depends on the happiness of others with whom they are connected. They found clusters of happy and unhappy people were visible in the network, and the relationship between people’s happiness extended up to three degrees of separation (for example, to the friends of one’s friends’ friends). People who were surrounded by many happy people and those who were central in the network were more likely to become happy in the future. Specifically, a friend who lives within a mile (about 1.6 km) and becomes happy increases the probability of a person being happy by 25% (95%, CI 1% to 57%). Similar effects were seen in co-resident spouses (8%, CI 0.2% to 16%), siblings who live within a mile (14%, CI 1% to 28%), and next-door neighbors (34%, CI 7% to 70%)^27^.

**Figure 1-:**
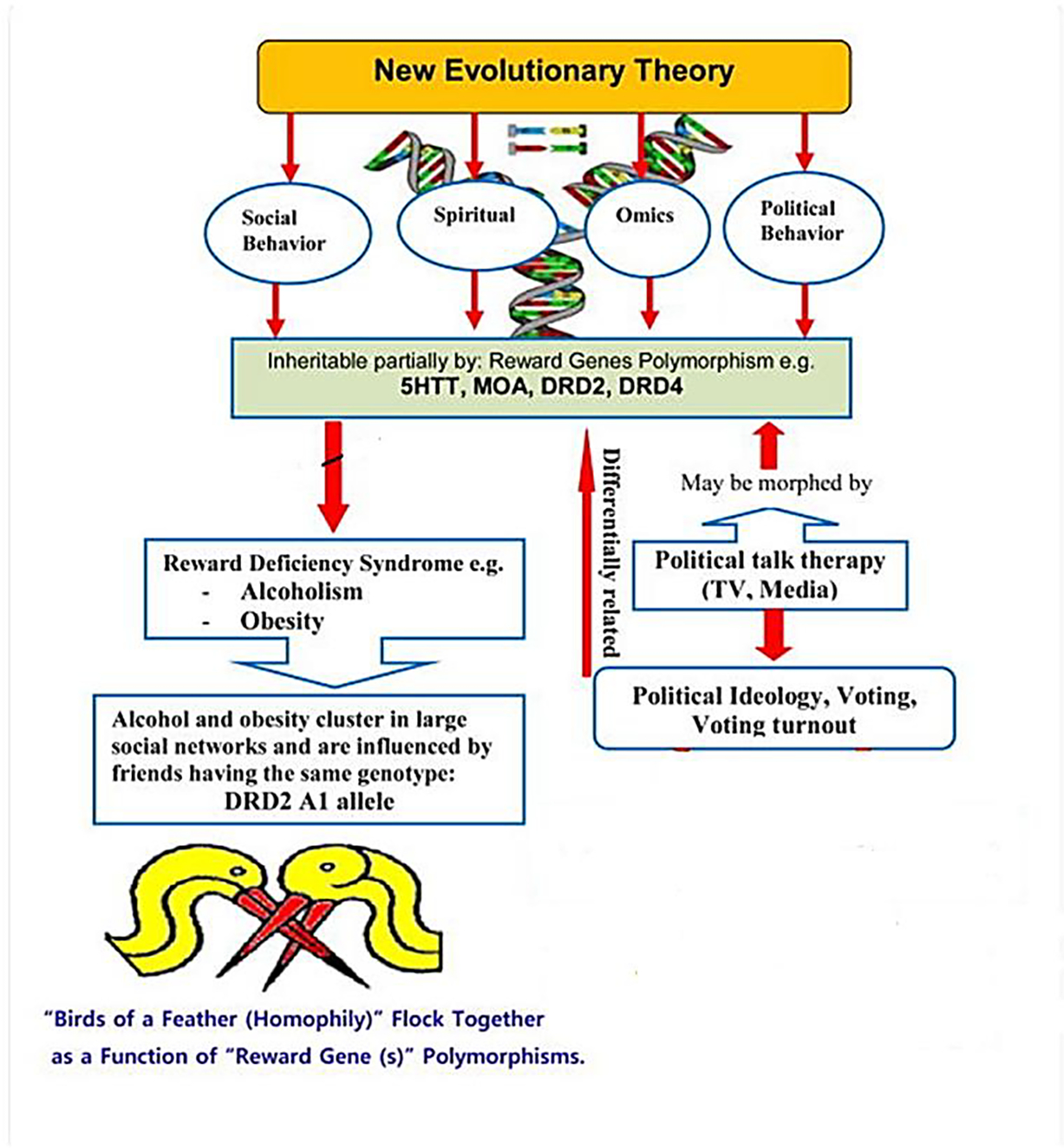
https://www.ncbi.nlm.nih.gov/core/lw/2.0/html/tileshop_pmc/tileshop_pmc_inline.html?title=Click%20on%20image%20to%20zoom&p=PMC3&id=3547641_nihms432296f2.jpg.

Using this development of a research model to define social networks, most recently, Fowler et al. correlated genotypes in friendship networks^28^. Using available genotype data derived from both the National Longitudinal Study of Adolescent Health and the Framingham Heart Study, they found that the DRD2 A1 allele is positively correlated (homophily) and CYP2A6 (SNP rs1801272) is negatively correlated (heterophily). These unique results show that homophily and heterophily occur on an allelic level. The results suggest that association tests should include friends’ genes and that evolution theories should consider that humans might, in some sense, be metagenomic concerning the humans around them (supporting the concept that *birds of a feather flock together* (Figure 1).

Germane, regarding reward genes and social networks, it is important to point out the original study by Blum et al. associating the DRD2 A1 allele with severe alcoholism^29^.

It is not surprising that Rosenquist et al. remarkably found that clusters of drinkers and abstainers were present in the network at all time points, and the clusters extended to three degrees of separation^30^. These clusters were not only due to the selective formation of social ties among drinkers but also seemed to reflect interpersonal influence. Accordingly, changes in the alcohol consumption behavior of a person’s social network had a statistically significant effect on that person’s subsequent alcohol consumption behavior. The behaviors of immediate neighbors and coworkers were not significantly associated with a person’s drinking behavior, but the behavior of relatives and friends was positively associated.

## The Preaddiction Construct: A Missing Piece In Understanding Addictive Behaviors

McLellan et al. urged the addiction biology and clinical field to consider the concept of Preaddiction as a missing term in the treatment of Substance Use Disorder(SUD)^31,32^. Recently, Sharafshah and associates presented data exploring the concept of “Pre-Addiction” within addiction biology through a comprehensive in silico analysis of 88,788,381 GWAS-based samples from 1,373 studies, identifying 18 significant genes (e.g., APOE with p-value= 1.0E-126) linked to Opioids, Pain, Aging, and Apoptosis pathways. It aims to correlate these genes with GARS, highlighting the most connected genes like MAOA, COMT, APOE, and SLC4A6 through a STRING-MODEL. The analysis expanded to 27 unique genes, emphasizing significant interactions with hsa-miR-16–5p and hsa-miR-24–3p, especially noting SLC6A4. Through PGx mining, 1,173 variant annotations were identified for these genes. Enrichment Analysis and Meta-analysis further solidified these findings, illustrating the pivotal role of dopaminergic pathways in connecting addictive behaviors and depressive symptoms, proposing reward deficiency syndrome (RDS) as the fundamental preaddiction phenotype, with pain, opioid dependence, aging, and apoptosis as critical endophenotypes^33^.

## Physiological Factors

Mammalian brains primarily operate on a motivational system with two types of motivation: like” and “want. Like” is regulated by opioid and brainstem systems, representing the pleasure experienced upon receiving a reward, while “want” is driven by the cortico-mesolimbic dopaminergic system, motivating the pursuit of rewards. Pleasure arises from intracellular signaling within adaptive chemical pathways of the reward system, directing attention toward fulfilling needs. Key brain regions in reward pathways associated with substances like alcohol, opiates, and cocaine include the nucleus accumbens (NAcb) and globus pallidus. Neurotransmitters such as dopamine, serotonin, enkephalins, GABA, and norepinephrine are involved in these pathways. Disruptions in this cascade can lead to chemical imbalances, triggering negative emotions, known as “reward deficiency syndrome,” which manifests as behavioral disorders due to deficiencies in the adaptive reward pathway. Drug addiction can both induce and exacerbate this syndrome^3^.

Van Staden and colleagues investigated neural regions sensitive to drug effects, which led to the identification of the supraesophageal ganglion, particularly the accessory lobes, as crucial targets for the psychostimulant effects of amphetamine and cocaine. Studying molecular responses in this region exposed to cocaine reward, they observed elevated cellular expression patterns of the immediate early gene CFOS, indicating recent neural activation. Crayfish conditioned with cocaine showed significantly higher CFOS expression in the accessory lobes compared to those conditioned with saline. Moreover, cocaine-treated individuals placed in novel environments displayed notable activation magnitude. This research on crayfish reveals surprising parallels with mammalian drug-related rewards, offering insights into addiction mechanisms. Despite their more straightforward neural structure, crayfish exhibit behavioral responses to human drugs of abuse^2^.

Research by Lewin-Epstien et al. has shed light on how the microbiome impacts the brain, behavior, and overall well-being of its host. Dysbiosis, an ongoing imbalance in the microbiome, has been associated with various chronic diseases, including addictive behaviors. The competition dynamics within the microbiome and mutual regulation between the host and microbiome may contribute to dysbiosis and worsen addictive behaviors. Significant disruptions to this ecosystem can drive the microbiome towards a composition that reinforces the new host state. This feedback loop exacerbates imbalances after disturbances, making it difficult to return to the initial balance and promoting relapse episodes, thereby prolonging addictions^34^.

## Psychological Factors

Psychoactive drugs elicit emotions that once signaled heightened fitness in mammalian evolution rather than just happiness. Positive emotions in ancient environments indicated increased fitness, such as successful foraging or breeding, while euphoria signaled high fitness levels, serving as a survival signal. Conversely, negative emotions were prevalent when fitness levels were low. Although many psychoactive substances produce euphoric sensations similar to those promoting fitness in ancient mammals, contemporary drug use may harm neural circuitry instead of providing anticipated fitness boosts^3^.

These drugs target ancient brain mechanisms, modulating incentive motivation within the nucleus accumbens and neural reward system. Modern drug addiction often stems from a misguided perception of increased fitness, leading to continued substance abuse despite adverse consequences. This paradox overrides adaptive behaviors, causing individuals to prioritize stimuli mimicking increased fitness over essential survival behaviors. Consequently, drug-seeking behaviors may compromise fitness, overshadowing the urge to fulfill basic needs and reducing viability as emotional focus shifts towards drug-seeking rather than survival^3^.

In contemporary society, drugs alleviating negative emotions may seem more beneficial than enduring ancient warning signals like pain and fever. However, the human body’s defense mechanisms have become hypersensitive, leading to unnecessary negative emotions as preventive measures against non-harmful stimuli. Drugs suppressing these defenses, like anxiolytics, may temporarily alleviate minor negative emotions but leave individuals vulnerable to more significant harm that contributes to decreased fitness.

Emotional dispositions closely correlate with problematic alcohol use. Negative emotions before alcohol consumption often led to drinking as a coping mechanism, with less control over consumption. Conversely, a positive disposition may lead to controlled usage to enhance mood. However, alcohol disrupts normal cognitive processes, suppressing negative emotions while reinforcing the habitual continuation of positive emotions. This can lead to relapse triggers in recovering alcoholics seeking to cope with negative feelings by drinking.

Drugs of abuse profoundly affect motivational states, enhancing the significance of associated stimuli and prioritizing interactions with novel stimuli. These effects closely resemble activating a primal emotion-Seeking drive- and generating rewarding signals. Even in invertebrates, psychostimulant drugs produce effects similar to those observed in mammals, which is crucial for the perception of reward within this system. Various addictive substances, including amphetamines, alcohol, and opioids like morphine and heroin, induce psychostimulant effects in crayfish resembling their effects in mammals. While different drugs may have varying effects, they can impact motivational and learning expressions differently^2^.

The core drivers of addiction manifest in altered and sometimes aberrant expressions of motivation and learning, traits that emerged early in evolution. Throughout evolutionary history, neural systems have evolved to optimize these functions for successful goal pursuit in an unpredictable environment. Exploration tendencies guide behavior to increase interactions with natural rewards essential for survival, such as food and water. These appetitive behaviors reflect the fundamental drivers of motivational states^2^.

## Societal Factors

The profound influence of environmental and psychological factors on the progression towards addiction is widely acknowledged. Various environmental conditions have been identified as exacerbating vulnerability, such as family dysfunction, a disadvantaged socioeconomic background, insufficient parental supervision, and widespread exposure to social drug use. These factors can contribute to the transition from a predisposition to substance abuse to full-blown addiction. Additionally, both acute and chronic stressors have been linked to substance abuse, with acute stress serving as a primary trigger for relapse among recovering drug addicts. Furthermore, readily available drugs can increase susceptibility, especially in economically disadvantaged communities, where factors like overcrowding and poverty are associated with higher rates of substance abuse. Moreover, exposure to successful individuals who use substances, whether through the media, peers, or family members, can influence the behaviors of children and adolescents. Similarly, the perception that smoking, drinking, or drug use is commonplace among peers can further encourage substance abuse^3^.

## Understanding Early Childhood Developmental Risk Factors

Early childhood, defined as the period from birth to eight years, is a time of rapid physical, cognitive, and emotional growth. During this formative phase, children develop crucial skills and patterns of behavior that can influence their susceptibility to addiction later in life. The brain’s plasticity during this period makes it particularly receptive to environmental influences, both positive and negative^35^.

A complex interplay of genetic, environmental, and psychological factors influences the risk of developing addictive behaviors. Identifying and mitigating these risk factors early can significantly impact a child’s future. Children with parents who misuse substances are at higher risk due to genetic predisposition, and modeling of addictive behaviors poses a key factor for the development of addiction^36^. Exposure to *Adverse Childhood Experiences (*ACEs), such as abuse, neglect, and household dysfunction, is strongly correlated with an increased risk of addiction^37^.

## Prevention

### EVIDENCE-BASED EARLY CHILDHOOD INTERVENTIONS:

Preventive interventions should be multifaceted, targeting various levels of influence from individual to community. Effective strategies include:

#### Parenting Programs –

Parenting programs such as parenting conferences, mentoring or coaching, and home visit programs that train first-time parents can improve parenting practices and reduce the risk of later substance use in children^38^.

#### Early Childhood Education –

High-quality early childhood education programs have been shown to promote cognitive and social development, thereby decreasing future substance abuse^39^.

#### Policy and Community Interventions –

Policies that reduce socioeconomic disparities and enhance community resources can create environments that support healthy development^40^.

### EARLY CHILDHOOD PROTECTIVE FACTORS

#### Secure Attachment-

Stable and nurturing relationships with caregivers foster emotional security and resilience^41^.

#### Social and Emotional Skills –

Teaching children emotional regulation, problem-solving, and social interaction can reduce vulnerability to addictive behaviors^42^.

## Treatment

In this paper, we propose the utilization of Awareness Integration Therapy as an efficient psychotherapeutic intervention for prevention, treatment, and relapse prevention.

Awareness Integration Therapy (AIT) is an evidence-based, multifaceted psychotherapeutic approach to enhance self-awareness, mitigate past traumas and psychological obstacles, and cultivate clarity and positive mindsets. It is crafted by synthesizing insights and techniques from diverse therapeutic models such as Cognitive Behavioral Therapy (CBT), Existential Therapy, Person-Centered Therapy, Emotion-Focused Therapy (EFT), Mind-Body Therapy (MBT), Eye Movement Desensitization and Reprocessing (EMDR), Hypnosis, and Mindfulness. AIT provides a comprehensive strategy by blending elements from these approaches, establishing a flexible framework to address the entirety of the human experience. This integration maximizes therapeutic effectiveness, facilitating enduring and transformative results for individuals undergoing treatment^42^.

Clinical investigations have revealed promising results regarding the efficacy of AIT as an applied model in addressing various psychological concerns that are underlying addiction and addictive behaviors. According to Zeine (2016), AIT has shown a 76% effectiveness rate in reducing depression, a 60% decrease in anxiety, a 43% improvement in self-esteem, and a 20% increase in self-efficacy^43^. Similarly, findings from a study conducted by Zeine et al. (2017) demonstrated notable reductions in depression (27.5%) and anxiety (37%) and increases in self-esteem (15%) and self-efficacy (13%) following a therapeutic six-hour AIT workshop^44^.

Moreover, utilizing AIT as a self-help module has shown promising outcomes among college students. Zeine et al. (2017) reported that participants experienced reduced stress levels and significant decreases in anxiety (21.72%) and depression (68%) following engagement with an AIT module^45^.

Case studies further support the effectiveness of AIT in addressing psychological distress. Madani and Zeine (2023) observed a 50% reduction in anxiety and a 60% increase in self-esteem following AIT intervention^46^. Similarly, Zarbakhsh and Zeine (2023) documented significant improvements in PTSD symptoms (66%), anxiety (75%), and depression (66%) among a transgender male college student undergoing AIT^47^.

Survey data of the “Foojan” app using AIT has also provided insights into the broad-ranging benefits of AIT across various domains. Zeine (2023) reported improvements in self-perception (64%), career aspirations (43%), intimate relationships (57%), relationships with children (55%), siblings (57%), in-laws (53%), mother (55%), and father (53%). These findings collectively underscore the multifaceted effectiveness of AIT in promoting psychological well-being and interpersonal functioning^48^.

AIT aims to reconcile fragmented aspects of the “Self” stemming from psychological trauma and to enhance self-awareness across past experiences to the present. This method embraces an individual’s life journey through an effective, open-ended approach, empowering Individuals to explore and grasp the interplay between their perception of the environment, interactions with others, and behavioral patterns using specified awareness skills. By engaging with both conscious and subconscious processes, AIT facilitates the reevaluation of irrational thoughts and emotions while assisting clients in developing valuable life skills.

Structured guidance for identifying fragmented aspects of the self and integrating them for harmonious and productive functioning is essential. Through a systematic process, Individuals differentiate between constructive and nonconstructive thoughts and mental schemas, thereby identifying deeply ingrained fundamental beliefs, emotions, and behaviors. Consequently, Individuals acquire new strategies to replace negative beliefs with constructive and beneficial principles. As AIT explores the interconnectedness of the self with various life domains such as relationships, family dynamics, career, finances, and spirituality, transformations extend across these realms.

The AIT model, a comprehensive approach, offers methodical assistance to integrate fragmented components of the self and facilitate harmonious and productive functioning. This multistep procedure identifies embedded underlying beliefs, emotions, and behaviors, distinguishing between constructive and nonconstructive thoughts and mental schemas. Subsequently, acquire fresh approaches to swap harmful ideas with positive, helpful ones. Transformations occur not only within the individual but also in relationships, family dynamics, career, finances, and spirituality, as AIT investigates the interconnectedness of the self with these domains, providing a sense of reassurance and confidence in its effectiveness.

For individuals struggling with substance abuse, the notion of living without their preferred drug often seems inconceivable, as addiction frequently replaces feelings of happiness, love, vitality, and connection. Motivating these individuals to change can stem from experiencing the consequences of continued substance use or from embracing more meaningful alternatives. However, relying solely on avoiding pain may lead to existential resentment and sorrow, perpetuating the cycle of relapse. While love and acceptance can be powerful catalysts for initiating sobriety, sustaining long-term recovery requires individuals to address their inner issues to resolve underlying emotional distress. Despite genetic predispositions and epigenetic influences, the Awareness Integration Theory offers a framework for individuals to evaluate their thoughts, emotions, and behaviors, fostering self-awareness and facilitating significant change.

Living with a person struggling with addiction is often a source of frustration, pain, and emotional exhaustion. Despite the family’s deep love and affection for the individual, there comes a point where their boundaries and judgments surface, which the addicted individual interprets as rejection. This sense of rejection exacerbates their feelings of shame and can trigger a return to substance use. Therefore, the approach of Awareness Integration Therapy (AIT) in psychotherapy, which offers unconditional acceptance and love while fostering awareness and accountability for one’s choices and outcomes in a non-judgmental manner, serves as a motivating factor for individuals to remain committed to their recovery journey, even in the event of relapse. For individuals with addiction who are married, have children, or live with parents, it is recommended to include family psychotherapy sessions to facilitate discussions, resolve conflicts, and establish boundaries among family members. Through the AIT process, family members can also gain insight into their behaviors and their impact on the individual with addiction. Each family member can then take responsibility and be accountable for their actions towards the individual with addiction, whether it involves enabling, using, abusing, nurturing, setting boundaries, or lacking thereof.

## AIT’s Six-Phase Intervention Model

Phase 1 focuses on cultivating awareness of the participant’s cognitive, emotional, and behavioral patterns in response to their external environment. This phase involves asking questions to uncover participants’ perceptions and attitudes towards others, revealing underlying belief systems that influence their worldview and interpersonal interactions.

Phase 2 focuses on three main objectives: helping the individual become aware of their subjective projections regarding others’ opinions of them, understanding how they attribute meaning to these perceptions and evaluating the impact of these internal projections on their life. Through targeted inquiries, clients explore their assumptions about how they are perceived by others, which is particularly beneficial for individuals dealing with heightened anxiety and social phobia.

Phase 3 focuses on fostering a reflective understanding of the participant’s identity by delving into core beliefs and feelings about themselves, reflecting on self-perception, emotions, and self-judgment, cultivating self-compassion, and promoting overall well-being.

Phase 4 involves a structured exploration of the connection between thoughts, cognitive patterns, emotional responses, and bodily sensations, focusing on negative core beliefs stemming from traumatic experiences. Participants assess how negative thoughts and emotions hinder rational thought processes and learn skills to manage emotions effectively. This phase facilitates the release, healing, and integration of fragmented aspects of the self, leading to a re-evaluation of self-belief systems and enhanced connectivity with the external world.

Phase 5 is forward-looking. It guides participants through visualization exercises, commits to cultivating a new self-concept, emphasizes nurturing a positive attitude, fosters agency in shaping perceptions and actions toward oneself and others, and teaches and implements new skills toward an addiction-free and functional life.

Phase 6 involves relapse prevention, structuring a functional value system aligned with intentions, emotions, and behaviors conducive to everyday life. Participants design their desired positive self, with visual aids such as collages providing consistent reassurance and guidance towards realizing their aspirations.

The above six phases will be conducted in all relevant areas of life that addiction has impacted, such as relation with drug or behavior of choice, body, career, finances, intimate relationship, family relation, sense of self, and spirituality^42^.

Traditionally, addiction has been seen as a response to deeper psychological, spiritual, and biological cues, encompassing developmental, pharmacological, and social elements. Recognition of addiction’s evolutionary origins is growing, with insights from addictive plant alkaloids and invertebrate behaviors. Repetitive behaviors triggering dopamine release may have initially served survival and reproductive functions but can now lead to addiction. This mismatch between ancient survival needs and modern stimuli can result in addictive behaviors and Reward Deficiency Syndrome^49,50^.

In sum, we are proposing an RDS-derived complementary measure of “preaddiction” that may provide further impetus for the optimal characterization of the construct, including its early detection, staging, and therapeutic management. The heuristic value of our proposal will be determined by its ability to account for specific clinical, genetic, and therapeutic aspects of the preaddiction phenomenon. Further research is warranted to uncover the distinctive aspects of RDS in addictive *vs*. other psychiatric pain and medical conditions and their interactions in the comorbid states^51^. In essence, our proposal relates to the importance of early genetic testing to identify preaddiction or RDS in children. As a therapeutic methodology for restoring neuroplasticity and recovery in genetic or behavioral addiction, AIT, a highly impactful non-invasive methodology, has been shown to have high efficacy results in brain remapping and activating to activate, inhibit, modify, or regulate neural activity^52,53^. AIT’s multifaceted result-oriented methodology is particularly instrumental in addiction recovery as its core principles center around the whole person, examining the core values, beliefs, cognition, and impact on behavior. Systematic reviews of AIT’s effectiveness have been examined, showing positive results in treating individuals’ foundational propensity to depression, anxiety, and addictive behavior^54^.

## Conclusion

The evolutionary perspective sheds light on the complexity of addiction. While some substances and behaviors may have benefited our ancestors, the availability of highly addictive stimuli today poses significant risks to the human species. Various factors, including genetics, epigenetics, psychology, social dynamics, and environmental cues, influence addiction. While once adaptive, addiction has become maladaptive in today’s environment of abundant rewards. Moving forward, interventions must address addiction’s evolutionary roots and disparities between past and present environments. Comprehensive strategies considering genetics, psychology, social factors, and environmental influences are crucial for effectively combating addiction’s impact on individuals and contemporary society. It is also prudent to consider how and if humans’ current adaptations may impact the addictive behaviors of future generations.

Preventing addiction in early childhood requires a comprehensive approach that addresses the myriad of factors influencing a child’s development. By fostering secure attachments, teaching social and emotional skills, and providing supportive environments through evidence-based programs, we can build resilience in children and reduce their risk of developing addictive behaviors. AIT therapeutical model, as was explained, can play a significant role in both preventative and treatment stages of addiction. An important aspect of the AIT model is its unique trauma-informed approach to treating individuals who intergenerationally have been exposed to traumatic events. Examining the horizontal, vertical, intersectional, and generational interconnectedness of trauma allows the therapist to use AIT to create a clear genogram of the ecology of addiction and preaddictive tendencies.
